# A pilot with computer-assisted psychosocial risk –assessment for refugees

**DOI:** 10.1186/1472-6947-12-71

**Published:** 2012-07-16

**Authors:** Farah Ahmad, Yogendra Shakya, Jasmine Li, Khaled Khoaja, Cameron D Norman, Wendy Lou, Izzeldin Abuelaish, Hayat M Ahmadzi

**Affiliations:** 1 School of Health Policy and Management, York University & Dalla Lana School of Public Health, University of Toronto, 4700 Keele Street, HNES Building, Room 414, Toronto, Ontario M3J 1P3, Canada; 2 Access Alliance Multicultural Health and Community Services, 340 College Street, Ste 500, Toronto, Ontario, M5T 3A9, Canada; 3 Research Coordinator for the project, Dalla Lana School of Public Health, University of Toronto, 155 College Street, Toronto, Ontario, M5T 3M7, Canada; 4 Dalla Lana School of Public Health, University of Toronto, 155 College Street, Toronto, Ontario, M5T 3M7, Canada; 5 Afghan Association of Ontario, 29 Pemican Crt, Toronto, Ontario, M9M 2Z3, Canada

**Keywords:** Computer-assisted, Health assessment, Community health centre, Pilot, Controlled trial, Refugee

## Abstract

**Background:**

Refugees experience multiple health and social needs. This requires an integrated approach to care in the countries of resettlement, including Canada. Perhaps, interactive eHealth tools could build bridges between medical and social care in a timely manner. The authors developed and piloted a multi-risk Computer-assisted Psychosocial Risk Assessment (CaPRA) tool for Afghan refugees visiting a community health center. The iPad based CaPRA survey was completed by the patients in their own language before seeing the medical practitioner. The computer then generated individualized feedback for the patient and provider with suggestions about available services.

**Methods:**

A pilot randomized trial was conducted with adult Afghan refugees who could read Dari/Farsi or English language. Consenting patients were randomly assigned to the CaPRA (intervention) or usual care (control) group. All patients completed a paper-pencil exit survey. The primary outcome was patient intention to see a psychosocial counselor. The secondary outcomes were patient acceptance of the tool and visit satisfaction.

**Results:**

Out of 199 approached patients, 64 were eligible and 50 consented and one withdrew (CaPRA = 25; usual care = 24). On average, participants were 37.6 years of age and had lived 3.4 years in Canada. Seventy-two percent of participants in CaPRA group had intention to visit a psychosocial counselor, compared to 46 % in usual care group [*X*^*2*^ (1)=3.47, *p* = 0.06]. On a 5-point scale, CaPRA group participants agreed with the benefits of the tool (mean = 4) and were ‘unsure’ about possible barriers to interact with the clinicians (mean = 2.8) or to privacy of information (mean = 2.8) in CaPRA mediated visits. On a 5-point scale, the two groups were alike in patient satisfaction (mean = 4.3).

**Conclusion:**

The studied eHealth tool offers a promising model to integrate medical and social care to address the health and settlement needs of refugees. The tool’s potential is discussed in relation to implications for healthcare practice. The study should be replicated with a larger sample to generalize the results while controlling for potential confounders.

## Background

More than eleven million people in the world are living outside their homeland as refugees [1]. Some of them receive resettlement opportunities offered by the developed countries, such as Canada which accepts 12,000 conventional refugees annually [2]. In Canada and elsewhere, the newly arrived refugees report multiple health and social needs due to the compounding effects of forced displacement, family separation, prolonged stay in over crowded camps, and acute material deprivation [3]. Nevertheless, refugees have resilience which is mediated by individual and socio-ecological resources.

Evidence show that provision of supportive environments such as employment and housing [4], ethnic networks and family cohesion [5,6], and opportunities to look forward rather than reiteration of painful past experiences [7,8] mitigate mental, physical and social health among refugees. The need to focus on social factors is also emphasized by the Canadian Collaboration for Immigrant and Refugee Health in producing the preventative health guidelines specific to this population [9]. Although special programs exist in the resettling countries, the social needs of refugees often receive limited attention compared to their medical needs [10]. Not surprisingly, refugees often remain unaware of several available social services (e.g., language classes, job training, social support groups and child care) to avail them in a timely manner [11]. This is a significant lost opportunity in terms of both individual and population health [12-14].

How can the social and medical services integrated for optimal settlement and integration of refugees? Perhaps, interactive eHealth tools could build bridges between the two in a timely manner. This is particularly relevant for the Community Health Centers where medical and social services co-exist. Recently, the authors developed a computer-assisted psychosocial risk assessment tool for Afghan refugees in Toronto, Canada and conducted a pilot study. This drew from previous work conducted with a similar but different tool for the general population visiting family medicine setting [15]. The primary objective of the new tool for refugees was to examine its potential to integrate medical and social services by using a proxy measure of patients’ intention to visit a psychosocial counselor. Several studies document that human intention to act is a strong predictor of actual action consistent with decades of scholarly work on behavior change theories [16-18]. The secondary objectives were to examine patients’ acceptance of the piloted tool and satisfaction with the visit.

## Methods

### Study site

The study was conducted in collaboration with Access Alliance Multicultural Health and Community Services in Toronto. This is a Community Health Centre (CHC) with a multidisciplinary model of care. The staff includes nurses, physicians, psychiatrists, dieticians, social workers, interpreters, peer-outreach workers, and settlement workers. The CHC provides primary health care, community outreach programs (e.g., peer support groups, language classes, and expressive arts programs), and settlement services to immigrants and refugees. The research ethics approval was obtained from the affiliated academic institution.

### Intervention

The study intervention was a touch-screen self-assessment survey which Afghan refugee patients completed on a touch-screen iPad in Dari/Farsi language while waiting to see their medical healthcare provider. The Computer-assisted Psychosocial Risk Assessment (CaPRA) survey had questions on psychosocial risks: substance use, exposure to personal violence, depressive symptoms, food and income insecurity, employment, social network, migration status, and coping. To reduce the social sensitivity, the survey also included questions on cardiovascular risks (e.g., physical activity, weight, diabetes, and hypertension) and road and home safety. The eHealth tool generated two tailored print-outs at the point of care. The recommendation sheet for patients summarized their disclosed risks in simple Dari/Farsi language. For the disclosed risks or concerns, tailored messages were printed to enhance patients’ self-esteem (e.g., no one deserves to be hit or there are ways to get your credentials evaluated) and to encourage discussions with the clinician and/or contacts with psychosocial counselor and community services. The risk-report for medical providers summarized patients’ risks with possible referrals. This was attached to the medical chart prior to the consult.

The development of CaPRA content involved multiple phases and a collaborative process. The team first identified the key psychosocial health issues for refugees by a literature review. In absence of specific guidelines for refugee health at that time, the team relied on community engagement process, overseen by an advisory group, to identify priority areas. We sought multiple perspectives by holding brainstorming sessions with healthcare providers (e.g., family physicians, nurse practitioners, social workers, and settlement workers) and representatives of refugee community and organizations serving Afghan refugees. This led to identification of the priority psychosocial risks for which services were also available at the collaborating site. Next, a preliminary version of the multiple-risk survey and messages were developed followed by translation and back-translation [19]. These were further refined for clarity and acceptance by conducting ten qualitative interviews with refugee clients and providers. The final paper-based versions were then converted into a computerized version in both English and Dari/Farsi languages. Prior to the pilot trial reported here, we tested the usability of iPad version with ten refugee clients and providers and made minor adjustments.

### Participants and procedures

All site physicians and nurse practitioners received details about the study and provided informed consent. The providers then attended a workshop, moderated by the collaborating clinician, on the risks included in the CaPRA tool. In the resettlement context of newcomer refugees, the discussion focused on the clinical guidelines and best practices for the risk identification, assessment and management. Providers were kept blind to the study’s main focus of intervention’s effect on patient intention to see a psychosocial counselor.

Afghan refugee patients were eligible to participate if they were over 18 years of age, could speak and read Dari/Farsi or English language, were eligible for federal or provincial health care program, and were visiting a participating provider. A bilingual research assistant (RA) approached the potential participants in the waiting room and applied eligibility criteria. If eligible and willing, interested patients received the study details in a separate room. Those who provided informed consent were randomly assigned to the CaPRA group (intervention) or the usual care group (control) with an allocation ratio of 1:1. Before recruitment, the randomization assignment was computer-generated by an off-site biostatistician using varying block sizes for each provider [20]. These patient assignments were sealed in opaque envelopes that were marked on the outside with a physician number and sequence number. The envelopes were opened by the recruiter after patients’ written consent. Afghan patients assigned to the CaPRA group completed the computer survey by using a touch screen iPad. The computer-generated risk-reports were attached to the patient’s medical chart. These patients also received a computer generated recommendation sheet. Patients assigned to the control group continued to receive usual care with no risk assessment before the consultation. Patients in both groups completed a paper-pencil Exit Survey after the visit. Each participant received $30 honorarium and a resource list for community-based services.

### Data collection and outcomes

Exit Survey collected information from all participant patients. The section on demographics had questions on age, gender, marital status, education, source of income, English fluency, number of years lived-in-Canada, and use of computers. The section on psychosocial health included questions on patient self-rated health, depression, exposure to violence, purpose of visit, provider discussion on psychosocial issues (i.e., mental health, stress, violence, income, work, language, job, or school), satisfaction with the received care, and patient intention to see a psychosocial counselor. Appointments were made by medical secretary for participants who expressed intention to see a counselor. Questions about patient acceptance of the tool were included for the CaPRA group.

The primary outcome of *patient intention* to visit a psychosocial counselor was measured by a single item (yes/no)*.* The secondary outcome of *patient acceptance* of the tool was measured in the CaPRA group by using a previously validated Computerized Lifestyle Assessment Scale (CLAS) with 12 items rated on a 5-point scale (strongly disagree, disagree, not sure, agree, strongly agree) [21]. The three subscales of CLAS are: 1) Benefits (6-item) which represents patient perceived benefits toward the quality of medical consultation and means of achieving them; 2) Privacy-Barrier (3-item) which covers patient concerns about information privacy; and 3) Interaction-Barrier (3-item) which represents concerns about potential interference in the interaction with the healthcare provider. The other secondary outcome of *patient satisfaction* with the visit was assessed by a 5-point scale (very unsatisfied, unsatisfied, neutral, satisfied, and very satisfied).

### Statistical analysis

As this study was a pilot trial, we aimed to recruit 50 refugees based on “Recommendations for Good Practice” by Lancaster et al for pilot randomized trials [22], consistent with others [23].

The Exit Survey data was analyzed using Statistical Package for Social Sciences (SPSS version 18). We executed descriptive statistics (proportions and means) and two-group comparisons using Chi-square and Student t-test. The two-group comparison was not executed for variables where participants could select more than one response (e.g., sources of income, and reason for visit). Some response categories were collapsed due to small sample size (e.g., use of computers).

## Results

**Figure 1:**
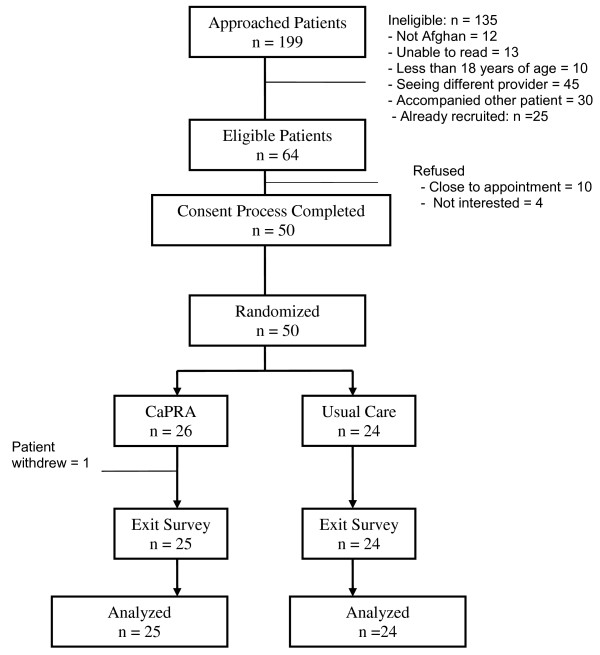
Patient Flow.

During July to October of 2010, 199 patients were approached in the clinic’s waiting room and 64 were found eligible (Figure 1). Out of 64 eligible patients, 20 men and 30 women provided informed written consent yielding a response rate of 78%. Fourteen eligible patients declined and the primary reason was lack of time because their appointment time was very close. One woman assigned to the CaPRA group did not feel well and withdrew.

### Participant demographics

Overall, the participants were 37.6 years of age (SD 13.7) and had lived 3.4 years (SD 1.3) in Canada. Majority of them were currently married or in a relationship (69%), had children (73.4%) and reported high school or less education (65%). Most of them were unemployed (84%) and received financial support from social welfare (54%), refugee assistance plan (10%), Ontario disability program (10%), employment insurance (2%), Canada pension plan (4%) and/or family (14%). Participants rated their English language abilities as ‘fair’ on a 5-point scale with a mean of 2.3 (SD .97). Almost one-third were using computers every day (30.6%) while a similar proportion (28.6%) had no previous experience.

On comparing the participants in the CaPRA (n = 25) and usual care (n = 24), statistically significant difference was found for the number of years lived-in-Canada (Table 1).

**Table 1:** Demographic and Health Characteristics

**Variable**	**CaPRA (N = 25)**	**Usual Care (N = 24)**	**P ValueX**^**2**^**or t-test**
Age, mean (SD)	41.1 (14.0)	33.9 (12.8)	0.07
Years lived in Canada, mean (SD)	2.9 (1.1)	3.9 (1.3)	0.005
Gender, Female % (n)	65.4 (17/25)	54.2 (13/24)	0.42
Currently in relationship, % (n)	58.3 (14/24)	79.2 (19/24)	0.12
Had children, % (n)	84.0 (21/25)	62.5 (15/24)	0.09
Highest level of Education, % (n)			
Up to high school	58.3 (14/24)	72.7 (16/22)	0.31
College/university (any)	41.7 (10/24)	27.3 (6/22)	
English reading/speaking*, mean (SD)	2.2 (.97)	2.4 (.97)	0.45
Computer use, % (n)			0.85
Everyday	28.0 (7/25)	33.3 (8/24)	
Twice a wk to once a month	40.0 (10/25)	41.7 (10/24)	
Not at all	32.0 (8/25)	25.0 (6/24)	
Sources of Income (select any), % (n)			-
Ontario Works (social welfare)	61.5 (16/25)	45.8 (11/23)	
Refugee Assistance Plan	11.5 (3/25)	8.3 (2/23)	
Ontario Disability Program	3.8 (1/25)	16.7 (4/23)	
Employment/ employment insurance	7.6 (2/25)	16.7 (4/23)	
Canadian Pension Plan	0.0 (0/25)	8.3 (2/23)	
Family support	11.5 (3/25)	16.7 (4/23)	
Self-rated Health*, mean (SD)	2.2 (1.1)	2.5 (1.1)	0.22
Physically abused in last 5 yr, % (n)	24.0 (6/25)	25.0 (6/24)	0.94
Depressive symptoms over last 2 weeks, % (n)	56.0 (14/25)	54.2 (13/24)	0.90
Reason for visit (select any), % (n)			
Routine physical	48.0 (12/25)	54.2 (13/24)	-
Follow up	60.0 (15/25)	29.2 (7/24)	
New concern	8.0 (2/25)	29.2 (7/24)	
Others	0.0 (0/25)	16.7 (4/24)	
Provider discussed psychosocial issues, % (n)	66.7 (16/24)	72.7 (16/22)	0.66
Satisfaction with the care received**, mean (SD)	4.4 (1.1)	4.2 (.9)	0.46

*Scale 1 to 5: poor; fair, good, very good, excellent.

**Scale 1 to 5: very dissatisfied, dissatisfied, unsure, satisfied, very satisfied.

### Participant psychosocial health

Overall, the participants rated their health as ‘fair’ on a 5-point scale with a mean of 2.3 (SD 1.1). In response to 2-item depression screen, a large number reported feeling down (69.4%) or a lack of pleasure (62.5%) during the last two weeks. Fifty-five percent (27/49) reported both of these symptoms; more among women (19/29) than men (8/20). One fourth of the participants (12/49) had experiences of personal violence during the last 5-years; more among women (9/29) than men (3/20). Participants identified the perpetrator as a stranger (22.4%) or someone they knew (11.9%). The most common reason for their visit on the day of recruitment was to have a routine physical checkup (51%) or a follow-up consult (40.8%). Majority of the participants (69.6%) reported that their provider discussed psychosocial issues during the visit. On comparing the two groups, participants were similar in the self-rated health, depressive symptoms and exposure to violence.

### Outcomes

For the primary outcome, the two groups were compared for the *patient intention to visit a psychosocial counselor.* Seventy-two percent of the participants in the CaPRA group showed intention to visit a psychosocial counselor compared to 46 % in the usual care group (Table 2) but the difference was not statistically significant [*X*^*2*^ (1)=3.47, *p* = 0.06].

**Table 2:** Patient Intention to Visit a Psychosocial Counselor Chi-Square Test

**Group**	**Intention to see a Psychosocial Counselor**		
	No	Yes	
	7	18	25
CaPRA	(28 %)	(72 %)	
Usual Care	13	11	24
	(54 %)	(46 %)	
	20	29	49

*X*^*2*^ (1) =3.47, *p* = 0.06.

For the secondary outcome of *patient acceptance*, participant scores were examined for each of the three CLAS subscales. Overall, participants had positive attitudes towards the CaPRA (Table 3). On a scale of 1 to 5, participants ‘agreed’ with the Benefits of the tool (mean = 4.0). Participant scores were in the middle of 5-point scale for the Privacy-Barriers (mean = 2.8) and Interaction-Barriers (mean = 2.8) indicating ‘unsure’ status. For the secondary outcome of *patient satisfaction,* no group difference was found between the CaPRA and usual care group and the mean score was 4.3 (SD 1.0) on a 5-point scale. When treating it as an ordinal variable, 84% of the participants in the CaPRA group and 74% in the usual care were ‘satisfied/ very satisfied’.

**Table 3:** Patient Acceptance

**CLAS Subscales**	**Mean (SD)**^**a**^
**Benefits**	**4.0 (.37)**
1. It would save the providers' time.	3.1 (1.2)
2. The computer is a good way to ask about social and emotional issues	4.4 (.71)
3. I would feel comfortable answering questions on a computer	4.6 (.50)
4. Computers-assisted risk assessment will help providers with questions on social and emotional health	4.2 (.55)
5. Computers-assisted health risk assessment can be trusted	4.0 (.62)
6. Providers will make better health assessments with such computer systems	3.8 (.87)
**Privacy-Barrier**	**2.8 (.74)**
1. I would worry about confidentiality when completing computer survey	2.8 (1.3)
2. I do not want certain information about me on the computer	3.0 (1.4)
3. Too many mistakes will be made with the computer-assisted risk assessment	2.5 (1.3)
**Interaction-Barrier**	**2.8 (.89)**
1. Providers would spend less time with patient	3.3 (1.3)
2. There will be loss of personal communication with a provider	2.8 (1.2)
3. I would find another provider with no such tool	2.1 (.99)

^a^ Scale 1 to 5: strongly disagree, agree, not sure, agree, strongly agree.

## Discussion

The findings of the pilot study suggest that a user-friendly, anonymous, self-administered iPad based touch-screen tool for patients in the waiting room of clinical settings can overcome barriers to psychosocial health-risk assessments in ways that better integrate medical and social services. The studied CaPRA tool positively influenced the intention of recent Afghan refugee patients to visit a psychosocial counselor. Further, the participants agreed with the benefits of the tool and did not necessarily perceive it as a barrier to interact with the clinicians or a barrier to their privacy of information. Notably, the use of tool kept the participants ‘very satisfied’ about the care they received and to the same level as the participants in the usual care. The results are discussed in relation to healthcare practice followed by field challenges and limitations.

The health challenges of the 21^st^ century needs innovative models of practice [24,25]. On one side, chronic and complex conditions are on the rise due to population aging and diversity. On the other, poor coordination across sectors is leading to inappropriate use of services and concerns about quality of care. Integration of services across sectors is one of the key healthcare reforms recommended by the World Health Organization [26]. This vision is also embraced by the health centers serving migrant populations [27]. However, these centres face many barriers to integrating care. Recent interactive and user-friendly eHealth tools could be used to meet the integration goal effectively. The eHealth model presented in this study enhanced attention of the medical providers and patients to the services available through psychosocial counselors - a step towards integration of medical and social care. Results suggest that providing patients with an anonymous, instantaneous self-assessment process with tailored recommendation sheet prior to their medical visit can promote self-reflection about psychosocial risks and trigger intention to receive care from a counselor. The CaPRA acceptance scores measured by the CLAS scale are very similar to those reported for English speaking patients who used such a tool in a family medicine clinic [19]. This enhances confidence in the transferability of this tool including different languages. We anticipate that the studied eHeath tool would contribute in the development of evidence-informed models of effective and integrated primary care provision.

The study findings also demonstrate the need to offer comprehensive care to newcomer refugees from Afghanistan, with attention to mental health issues. The high rates of depressive symptoms, low self-rated health and exposure to violence in the last five years are notable in our study and consistent with other studies with Afghan refugees [28-30]. It is also important to acknowledge that compromised mental health and experiences of violence are culturally sensitive issues. Thus, provider capacity in culturally sensitive care is an essential element for effective psychosocial risk assessments in primary care settings whether it is computer-assisted or not. To this end, our collaborative approach facilitated the engagement of multiple providers at the partnering agency in ways that actively reflected on sensitivity and stigma associated with addressing these issues. Provider competency is reflected in high and similar level of patient satisfaction for both the groups. In addition, privacy issues (including the availability of private rooms to use the tool), effective referral process, clarifying provider perspectives about computer literacy among clients, training for providers, medical secretaries and other clinic staff, and ongoing monitoring and evaluation are important considerations for routine use of this eHealth tool.

### Limitations and challenges

The study findings should be interpreted in light of the design limitations and the context of place and time. The study was a pilot trial with a select group of refugees visiting a single Community Health Centre. This limits the generalizeability to all refugee groups or newcomers. Yet, participants’ response rate of 78% indicates its likely acceptance across vulnerable communities. The interpretation of results warrant caution due to small sample, differences between two groups and volunteer bias of the participant providers. Although statistically significant difference in the demographics of two groups was found only for the number of years lived-in-Canada, we noted some differences in age, gender and level of education. Future research with a larger sample size is needed to allow control of potential confounders. Further, a follow-up component should be included in future designs to assess the impact on patients’ quality of life overtime once they access the referred or suggested psychosocial services.

It is also important to review some field challenges to inform future work. The study applied narrow eligibility criteria (e.g., exclude new patients) and focused on Afghan refugees due to resource limitations in developing a language specific tool. Consequently, few patients were eligible out of nearly two hundred approached in the waiting room. The focus on one sub-group of patients also inhibited the collaborating site, in compliance with the health information and privacy act, to send any pre-visit information letters to patients about the study to encourage early arrivals. Further, a number of practicing clinicians moved to a different clinic along with their patients during the recruitment phase. Although the clinic hired new providers and accepted new patients within a short time, the study recruitment criteria did not allow inclusion of new patients or those seeing new providers. This lengthened the recruitment time. Future research should consider multiple health centers and broader eligibility criteria to address such field challenges. Future models could explore offsite completion of the computer-assisted health risk assessments. However, assessment of socially sensitive issues might not suit non-synchronized approach because provider’s prompt response might be needed. Finally, additional technological advances should also be incorporated, such as linking the printouts to electronic medical records to reduce the documentation burden on the clinician and the use of voice to facilitate reading by patients and to address literacy issues.

## Conclusion

Interactive computer-assisted health-risk assessment tool in the waiting room of primary care settings is a promising approach to bridge health and social care for refugees and newcomers. In Labonte words “…universal programs without some targeting within them (some deference to greater disparity, greater need, greater historic exclusion) can heighten inequalities in outcomes because of who is better able to avail of such programs” [31]. We anticipate that wide adoption of such eHealth mediated model of care could contribute in addressing health disparities.

## Competing interest

The authors declare that they have no competing interests.

## Authors’ contributions

All authors made signification contributions: conception (FA, YS, JL, CN), design (FA, YS, JL, CN, WL, KK, IL, HMA), acquisition of data (FA, YS, JL, KK), analysis (FA, KK, WL), interpretation (FA, YS, JL, CN, WL, KK, IL, HMA), first draft of the manuscript (FA, YS, JL, KK) and critical review, revisions (FA, YS, JL, CN, WL, KK, IL, HMA). All authors read and approved the final manuscript.

## Pre-publication history

The pre-publication history for this paper can be accessed here:

http://www.biomedcentral.com/1472-6947/12/71/prepub
